# Increased Permeability of the Aquaporin *So*PIP2;1 by Mercury and Mutations in Loop A

**DOI:** 10.3389/fpls.2016.01249

**Published:** 2016-08-30

**Authors:** Andreas Kirscht, Sabeen Survery, Per Kjellbom, Urban Johanson

**Affiliations:** Department of Biochemistry and Structural Biology, Center for Molecular Protein Science, Lund UniversityLund, Sweden

**Keywords:** aquaporin, water channel, major intrinsic protein, *Spinacia oleracea*, tryptophan fluorescence

## Abstract

Aquaporins (AQPs) also referred to as Major intrinsic proteins, regulate permeability of biological membranes for water and other uncharged small polar molecules. Plants encode more AQPs than other organisms and just one of the four AQP subfamilies in *Arabidopsis thaliana*, the water specific plasma membrane intrinsic proteins (PIPs), has 13 isoforms, the same number as the total AQPs encoded by the entire human genome. The PIPs are more conserved than other plant AQPs and here we demonstrate that a cysteine residue, in loop A of *So*PIP2;1 from *Spinacia oleracea*, is forming disulfide bridges. This is in agreement with studies on maize PIPs, but in contrast we also show an increased permeability of mutants with a substitution at this position. In accordance with earlier findings, we confirm that mercury increases water permeability of both wild type and mutant proteins. We report on the slow kinetics and reversibility of the activation, and on quenching of intrinsic tryptophan fluorescence as a potential reporter of conformational changes associated with activation. Hence, previous studies in plants based on the assumption of mercury as a general AQP blocker have to be reevaluated, whereas mercury and fluorescence studies of isolated PIPs provide new means to follow structural changes dynamically.

## Introduction

Aquaporins (AQPs) are common in all domains of life and facilitate permeation of a wide range of small polar molecules through biological membranes (Abascal et al., 2014). AQPs are generally found as homotetramers, where each monomer constitutes a separate pore formed by six transmembrane helices and two short helices that are connected in the middle of the lipid bilayer at their N-termini (Fu et al., 2000; Sui et al., 2001; Törnroth-Horsefield et al., 2006; Horsefield et al., 2008; Kirscht et al., 2016). Members belonging to the plant subfamily of plasma membrane intrinsic proteins (PIPs) are specifically permeable to water and show a more strict amino acid sequence conservation than AQPs in other subfamilies (Danielson and Johanson, 2010). Function and abundance of these proteins is tightly regulated and the high number of isoforms suggests a highly redundant system for water homeostasis (Johanson et al., 2001; Alexandersson et al., 2005, 2010). Structure and regulation of one particular member of the PIP subfamily from spinach (*Spinacia oleracea*), *So*PIP2;1, which constitutes a dominating integral protein of the plasma membrane has been thoroughly studied (Johansson et al., 1996, 1998; Kukulski et al., 2005; Törnroth-Horsefield et al., 2006; Nyblom et al., 2009; Frick et al., 2013).

The structure of *So*PIP2;1 was solved in different conformations, which elucidated a general molecular gating mechanism for the regulation of PIPs, including some specific elements only relevant for members of the PIP2 subgroup (Törnroth-Horsefield et al., 2006). The gating of the pore is the result of conformational changes on the cytoplasmic side of the membrane and is controlled in several different ways, including, pH changes, binding of calcium and a PIP2 specific phosphorylation. The second cytosolic loop, Loop D, undergoes a major conformational change and its stabilization in certain positions is increasing the probability of an either open or closed conformation. In the closed conformation, unphosphorylated serine 274, situated close to the C-terminus, occupies a place near the tetrameric center of the protein while the preceding region resides in a grove between the monomers. When this positioning is impossible, e.g., by phosphorylation of serine 274, the C-terminus becomes unordered and the place earlier occupied by this residue is instead taken by the carbonyl oxygen of leucine 197 from the neighboring monomer, resulting in the unblocking of the pore in this monomer.

Already in early protein preparations, it became clear that interactions between monomers are strong in *So*PIP2;1 tetramers (Johansson et al., 1996). Even during separation by a standard SDS-PAGE, which denatures most proteins, a large fraction of the protein is found to stay as dimers and tetramers (Karlsson et al., 2003). PIPs differ from all other plant AQPs by a highly conserved cysteine in the first extracellular loop (loop A). The conserved cysteines (C69 in *So*PIP2;1) from all four monomers are located at the tetrameric center (Törnroth-Horsefield et al., 2006). Based on low resolution structures, it had been suggested that the reason for the conservation stems from the necessity to stabilize the PIP tetramers by fostering hydrogen bonds or complexing a metal ion (Kukulski et al., 2005).

To analyze the nature of possible interactions of the conserved cysteine (C69) in the tetramer, we probed possible disulfide bridges by addition of reducing agents. For further analysis, we created mutants and purified the heterologously expressed proteins. In this study, we measured stability changes for cysteine 69 mutants and compared these to their kinetic properties to elucidate the structural and functional basis for the strong evolutionary conservation of this amino acid residue. In addition we show that mercury – generally regarded as a blocker of AQPs – is activating *So*PIP2;1 and concentration-response experiments suggest that quenching of tryptophan fluorescence reports on conformational changes associated with the activation. Our findings of disulfide bridges and mercury activation are largely consistent with results recently published by other groups (Bienert et al., 2012; Frick et al., 2013).

## Results

### A Conserved Cysteine Forms Disulfide Bridges between Monomers

**FIGURE 1:**
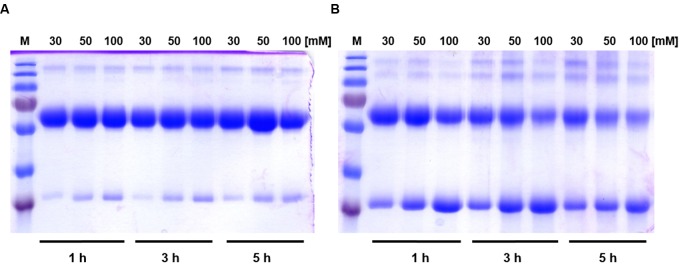
**The effect of reducing agents on relative amounts of dimers and monomers of *So*PIP2;1.** **(A)** Incubation of *So*PIP2;1 with DTT. The concentration of DTT and the incubation time are shown at the top and at the bottom of the gel, respectively. It is evident from the gel that DTT is unable to completely reduce dimers into monomers, even after 5 h incubation with 100 mM DTT. **(B)** Effect of incubation of *So*PIP2;1 with β-mercaptoethanol. The concentration of β-mercaptoethanol and the incubation time are shown on top and bottom of the gel, respectively. The results show that β-mercaptoethanol is more effective than DTT, but it still fails to reduce the dimers into monomers completely. M = molecular weight marker (from top 250, 130, 100, 70, 55, 35, and 25 kDa).

**FIGURE 2:**
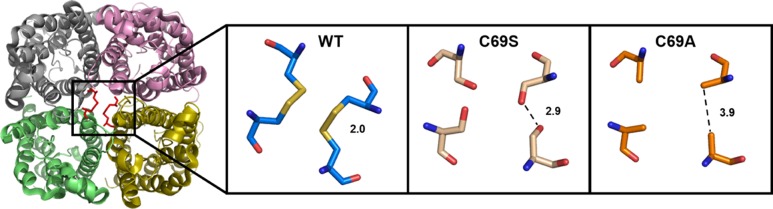
**Extracellular view of the *So*PIP2;1 wild type (WT) tetramer with the expected disulfide bridges at the center **(Left)**.** Close up on C69 with distances between cysteines (PDB ID: 2B5F) or modeled residues in mutant proteins with this cysteine replaced by serine **(Middle)** or alanine **(Right)**.

**FIGURE 3:**
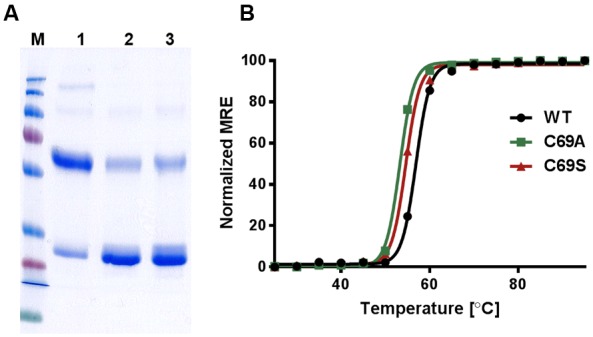
**Stability of WT *So*PIP2;1 and the mutants C69A and C69S.** **(A)** The integrity and purity of WT and mutant protein after Ni-NTA affinity chromatography were assessed by SDS-PAGE. 1 = WT *So*PIP2;1, 2 = C69A, 3 = C69S. The purified WT *So*PIP2;1 exists as tetramers under native conditions. Under standard denaturing conditions (SDS-PAGE), the WT *So*PIP2;1 mainly runs as a dimer. In case of cysteine mutants, the monomeric form is predominant. This indicates that the cysteine residue in loop A is involved in disulfide bond formation between two PIP monomers. M = molecular weight marker (from top 250, 130, 100, 70, 55, 35, 25, 15, and 10 kDa). **(B)** Thermal denaturation of *So*PIP2;1 recorded by CD spectrometry. The midpoint of the two-state unfolding transition is slightly higher for the WT (56.9°C) compared to the mutants (C69S 54.6°C; C69A 53.4°C). The midpoints are based on one experiment. The normalized mean residual ellipticity (MRE) at 222 nm is plotted as a function of temperature.

To discern inter-monomeric interactions of *So*PIP2;1 further, we decided to study structural and functional properties of isolated wild type (WT) and mutant protein. Therefore, we overexpressed *So*PIP2;1 in *Pichia pastoris* and performed different stability tests of solubilized and purified protein. The isolated WT protein was incubated for various length of time with different reducing agents prior to analysis by SDS-PAGE, in order to monitor any effect of potentially disrupted disulfide bridges on the migration pattern in acrylamide gels (**Figure 1**). The relative amount of SDS resistant dimer in the gel could be decreased by DTT and β-mercaptoethanol. The latter was more effective, but none of the reducing agents could increase the monomer-to-dimer ratio further after the first hour of incubation. Surprisingly, the split of the dimer was not increased above 100 mM DTT and the final ratio of monomer to dimer was less than 1/10, while β-mercaptoethanol decreased the dimers by 50% at most (not shown). Either this reflects the equilibrium between monomeric and dimeric fraction when all disulfide bonds are reduced, mainly stabilized by hydrophobic interactions (Törnroth-Horsefield et al., 2006), or some of the SDS solubilized dimers have disulfide bonds that are inaccessible so that they cannot be reduced under these conditions. The latter is supported by the apparent higher efficiency of β-mercaptoethanol which has about half the molecular weight compared to DTT. Adding urea to the reduction step increased the monomeric fraction (not shown). However, not even incubation for 1 h with 8 M urea and 300 mM DTT and resolving the samples on an 8 M denaturing urea gel was sufficient to completely remove the multimeric bands (**Supplementary Figure 1**). From a physical and evolutionary point of view it is of interest if the stability of the protein is changed upon removal of the cysteine. Two mutants, substituted at cysteine 69 with serine (C69S) or alanine (C69A) (**Figure 2**), were expressed and purified and their oligomeric patterns (as shown by standard SDS-PAGE) were compared to the WT protein (**Figure 3A**). Although dimeric bands were still visible in both mutants, they were significantly weaker as compared to the WT protein, which was expected from the destabilizing effect of reducing agents on dimers of the WT protein. In order to compare the thermodynamic stability of WT *So*PIP2;1 and the two cysteine 69 mutants, we performed thermal denaturation circular dichroism (CD) spectroscopic measurements. A thermal denaturation curve was constructed based on the normalized mean residual ellipticity (MRE) at 222 nm (**Figure 3B**). The midpoint of transition in WT (56.9°C) was slightly higher in comparison with the mutants (54.6°C for C69S and 53.4°C for C69A), suggesting that the interactions at this position do not contribute much toward the thermodynamic stability, which is expected since the contribution of disulfide bonds to protein stability is kinetic rather than thermodynamic (Clarke and Fersht, 1993). Considering the high stability of *So*PIP2;1 in lipid bilayers (Plasencia et al., 2011), it is not clear if the relatively small additional thermal stabilization due to the disulfide bonds is sufficient to explain the strict conservation of this cysteine.

### Water Permeability Is Increased by Mutations in Loop A

**FIGURE 4:**
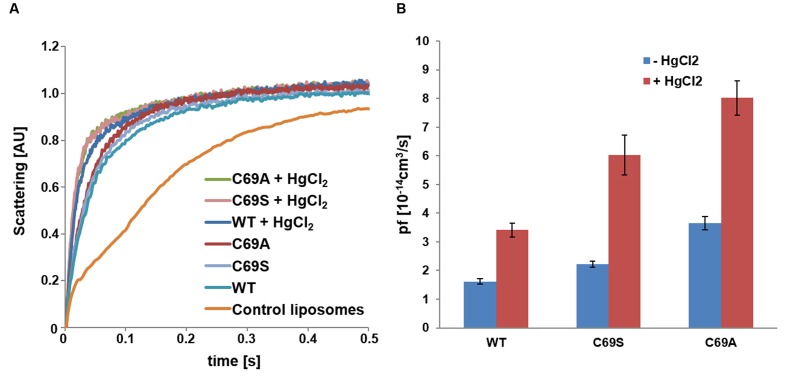
**Water conductivity of liposomes measured by stopped-flow spectrometry.** **(A)** Change in scattering at 500 nm over time plotted for *So*PIP2;1 WT and C69 mutant proteoliposomes as compared to empty liposomes. Each plot represents an average of 10–17 curves. **(B)** Monomeric permeabilities without mercury (blue) and with 1 mM mercury (red). The rates were obtained from single exponential fits of 10 or more curves; error bars present their standard deviations. The water conductivities were higher for the mutants as compared to the WT protein and mercury increased the *p*_f_-values of all proteins by a similar factor (2.1–2.7). The rate constants and the *p*_f_-values are provided in **Supplementary Table 1**, while the statistics for comparisons of the *p*_f_-values are reported in **Supplementary Table 2**.

To investigate if the disulfide bonds have an effect on function all three proteins were reconstituted in proteoliposomes to examine their kinetic properties. Since the activity assays confirmed functional proteins (**Figure 4A**), we assessed their permeabilities. The protein content in the liposomes was estimated by quantitative western blots and the monomeric permeabilities, the *p*_f_-values, were calculated. Surprisingly, the mutant C69S shows increased water permeability (*P* < 0.001) and this is even more pronounced in the C69A mutant (*P* < 0.0001; **Figure 4B**). The location of this residue immediately raises the question about water transport through the central pore. This scenario would be consistent with the observed larger enhancement of water permeability when cysteine is exchanged with an amino acid residue smaller than serine. Alternatively the increase is purely caused by destabilization of the dimer, where the alanine mutant would not be able to form hydrogen bonds like the serine mutant, which may partially compensate for the missing disulfide bridges.

### Mercury Increases Water Permeability and Quench Intrinsic Tryptophan Fluorescence

**FIGURE 5:**
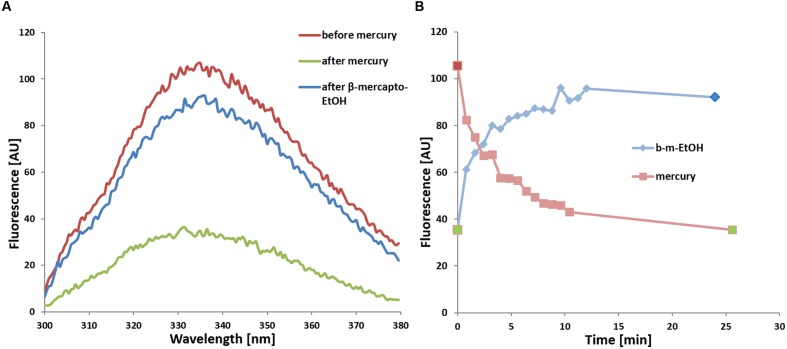
**(A)** Quenching of tryptophan fluorescence of WT *So*PIP2;1 upon addition of 200 μM mercury (green) and subsequent reversal by addition of 1 mM β-mercaptoethanol (blue) in comparison to untreated protein (red). The fluorescence at 335 nm from excitation at 280 nm is lowered by two thirds. **(B)** Time dependence of fluorescence quenching upon addition of 200 μM HgCl_2_ (light red) and recovering of fluorescence upon subsequent addition of 1 mM β-mercaptoethanol to the same sample (b-m-EtOH, light blue). The individual maximum of every spectrum are plotted, where each spectrum was recorded at a scanning speed of 100 nm/min (48 s per spectrum + 1 s delay). The spectra in **(A)** correspond to the three time points marked in the same color in **(B)**: before addition of HgCl_2_ (red), after 25 min incubation with HgCl_2_ but before addition of β-mercaptoethanol (green; replotted at time zero), and at the endpoint after the incubation with β-mercaptoethanol (blue).

**FIGURE 6:**
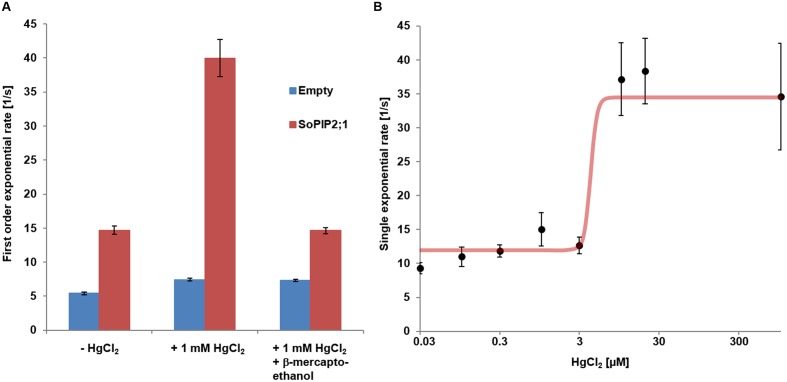
**Mercury activation of WT *So*PIP2;1.** **(A)** Reversibility of mercury activation first order exponential rates of water conductivity from stopped-flow experiments. Mean rates (±SD) are presented for proteo-liposomes (red) and control liposome (blue). Activation of proteo-liposomes treated with mercury can be reversed by subsequent incubation with 2 mM β-mercaptoethanol. Statistics for comparisons of the rate constants are reported in **Supplementary Table 3** and numerical values for mean rates and SD are found in **Supplementary Table 4**. **(B)** Dose response curve of mercury activation. The curve presents a Hill coefficient around 11 and an EC_50_ for mercury of 4.2 μM.

**FIGURE 7:**
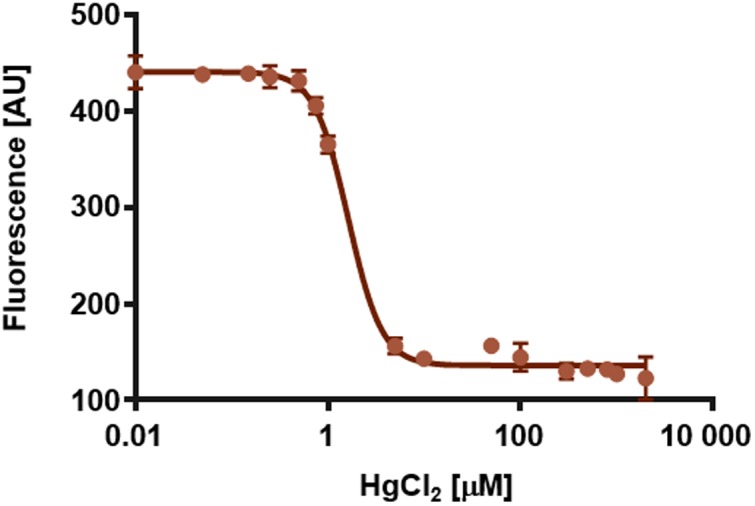
**Quenching of the intrinsic tryptophan fluorescence of *So*PIP2;1 at 335 nm as function of the logarithm of the HgCl_2_ concentration.** The calculated EC_50_ is 1.6 μM and the Hill slope is -2.5. Averages of three replicates and standard errors are plotted.

If some water permeation is independent of the pore in the monomer, it could be measured by eliminating the conductivity of the monomeric pore. Beside reported pH and Ca^2+^ dependent gating, mercury is a well-known inhibitor of AQPs (Preston et al., 1993; Hukin et al., 2002). To our surprise, mercury instead increased the permeability of *So*PIP2;1 proteoliposomes (*P* < 0.0001; **Figure 4**). If this effect is a result of conformational changes, there is a chance to observe mercury binding by CD measurements or by monitoring the intrinsic tryptophan fluorescence of *So*PIP2;1. The CD spectrum of *So*PIP2;1 in detergent micelles did not change significantly upon the addition of mercury (data not shown). However, the tryptophan fluorescence was altered and quenched by a factor of three after an incubation with 200 μM mercury chloride (**Figure 5**). Moreover, the quenching could be reversed by chelating mercury with β-mercaptoethanol, supporting that it is caused by reversible conformational changes rather than denaturation of *So*PIP2;1. This is in accordance with the kinetic experiments, where mercury activation can be reversed by addition of β-mercaptoethanol (**Figure 6A**). It should be noted that the concentrations of reducing agent used for the reversal of quenching and activation, 1 and 2 mM, respectively, are much lower than the levels required to partially reduce the disulfide bridge of the dimers. Thus given the modest increase in activity of the C69S mutant completely lacking the disulfide bridge, any increase due to reduction of cysteine 69 is expected to be small. This is consistent with the more or less identical rates of untreated proteoliposomes and the activated and reversed ditto. To investigate the concentration dependency of the activation stopped-flow experiments were done and the EC_50_ for mercury was calculated to be around 4 μM (**Figure 6B**). Interestingly, a concentration-response curve of the quenching of tryptophan fluorescence gives an EC_50_ value of 1.6 μM (**Figure 7**). Thus the quenching in micelles may be caused by conformational changes associated with activation in proteoliposomes.

### Activation by Mercury and by Loop A Mutations Are Additive

Cysteine 69 mutants present an increased water transport compared to the WT, which might be explained by opening of a tetrameric central pore or by indirect effects on the structure of the monomeric pore. WT *So*PIP2;1 can be activated by mercury, but are the cysteine mutants also affected by mercury? Analysis of stopped-flow data, showed that the activation by mercury and the increase of permeability by mutations were not overlapping, but additive (**Figure 4B**). While the *p*_f_ of the WT permeability changes from 1.6 ± 0.1 to 3.4 ± 0.2 [10^-14^ cm^3^/s], the *p*_f_ of alanine and serine mutants are amplified by about the same factor, rising from 3.6 ± 0.2 to 8.0 ± 0.6 and 2.2 ± 0.1 to 6.0 ± 0.7 [10^-14^ cm^3^/s], respectively. This suggest that the mercury activation is independent of the increase in water permeability caused by the mutations in loop A.

## Discussion

This work was originally initiated to describe intermonomeric interactions and their effects. The effect of reducing agents on the oligomeric pattern in SDS-PAGE indicated the existence of disulfide bonds that stabilize protein dimers. This was also concluded in studies mutating corresponding cysteines in the tetrameric center of various PIP2s from maize (Bienert et al., 2012). Additional to the more detailed analysis of disulfide bridges in the tetrameric center, we discovered that our preparation of *So*PIP2;1 was not maximally permeable under our standard conditions. It should be noted that the obtained *p*_f_-values here (1.6–8.0 [10^-14^cm^3^s^-1^]) are in the same range as has been estimated for other AQPs using *Xenopus* oocytes (0.2–24 [10^-14^cm^3^s^-1^]; Yang and Verkman, 1997) and correspond to a passage of more than 10^11^ water molecules per second through each monomer of *So*PIP2;1. Although activation of a water specific channel by mercury is unlikely to have a physiological role, the molecular details of its binding and conformational changes may still be relevant for our understanding of physiological gating mechanisms.

### Increased Permeability in Mutants and by Mercury

Our results suggest that the mechanism for activation by mercury is independent of the enhancement of water permeability caused by the mutations in the A loop. A simple mechanistic explanation of this would be that permeation through the monomeric pore is increased in the mutants and this is amplified by mercury by stabilization of the gate in an open conformation. However, looking at the structure it is not obvious how a mutation of cysteine 69 could achieve this, but it cannot be excluded that the release of loop A is resulting in a relaxation at the selectivity filter and thereby a higher permeability. If instead also the central pore of the mutant proteins permeates water, how would that be enhanced by mercury binding? Possibly in the same way mercury may support the open conformation of loop D at the monomeric pore; the C-terminal end is removed from its position in the inter-monomeric space. If the highest energy barrier for water permeation through the central pore is caused by interactions between the four C-termini, a destabilization of the C-terminal regions would increase the water leakage through this hypothetical pore. One should keep in mind that these C-terminal interactions are not structurally supported as the last modeled amino acid residues in the closed structures are still too far from the center. Thus a possible contribution of the central pore to the increased permeability remains speculative.

### Tryptophan Fluorescence

**FIGURE 8:**
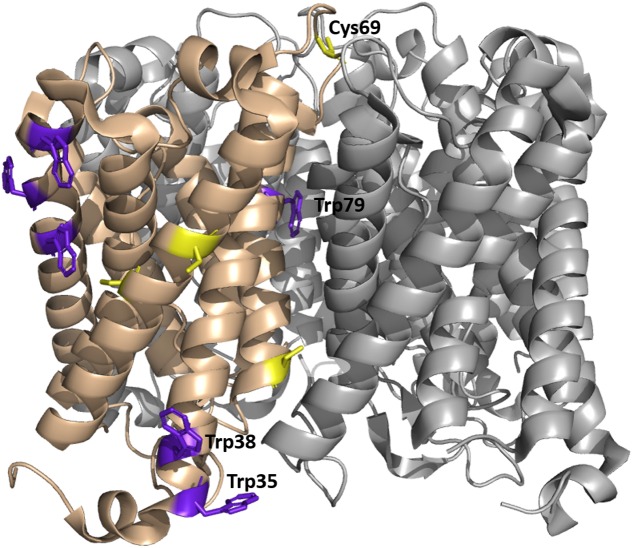
**Tetrameric structure of WT *So*PIP2;1 in a closed conformation (PDB ID 1Z98) showing positions of tryptophan and cysteine residues.** Each monomer exposes six tryptophans on its outside (purple); four of them (left in the picture) are expected to be in a non-polar environment (detergent micelle in buffer or a lipid membrane), one (tryptophan 79) is positioned in the tetrameric center and only tryptophan 35 is exposed to mobile waters, sitting at the beginning of helix 1 and close to the end of helix 6 and the C-terminus of the next monomer. Four cysteines are present in each monomer, two (left in the picture) are situated close to the tetramer equator, one is positioned in the monomer interface close to the cytosolic side, and cysteine 69 is highly accessible at the periplasmic side of the tetrameric center.

The observation that both activation and quenching of tryptophan fluorescence can be triggered by the same ligand, mercury, may suggest that these two effects are not independent but caused by structural rearrangements initiated from the same binding site. So where is the mercury binding and which conformational changes lead to a fluorescence drop of 2/3? If all six tryptophans of the monomer have the same quantum yield, at least four of them must be quenched. That involves a huge structural change of the protein or putatively a rearrangement of the micelle. Restructuring the micelle of a membrane protein is thought to denature the protein, which is unlikely here, as quenching (**Figure 5**) and increase in activity (**Figure 6A**) are both reversible, and no denaturation was indicated in CD measurements or by precipitating protein (data not shown). Fluorescence intensity of tryptophans can vary greatly depending on their environment (Lakowicz, 2006). Therefore, it appears more likely that one or two tryptophans with high quantum yields, located in a hydrophobic/hydrophilic interface, are responsible for the observed quenching of fluorescence by mercury. According to analyses of structural parameters tryptophans can be classified into five spectral-structural classes (Shen et al., 2008). The observed emission maximum at 335 nm would suggest that Class I and possibly Class II tryptophans dominate the fluorescence spectrum of *So*PIP2;1. This automatic analysis of PDB-files is most likely not applicable for tryptophans that are expected to interact with detergents in a micelle, since the micelle is not included in the structure file. However, it should be relevant for buried residues, like tryptophan 79 situated at the center of the tetramer (**Figure 8**). Based on the closed tetrameric structure (4JC6, omitting detergents or metal atoms) this residue belongs to Class S with an expected emission maximum range between 321 and 325 nm, and is therefore not likely to contribute much to the intensity at 335 nm.

As speculated above the mercury activation may be achieved by stabilizing an open conformation and interestingly the positions of tryptophans 35 and 38 differ in the open and closed structure (Törnroth-Horsefield et al., 2006). In the closed structure tryptophan 35 is potentially in a hydrophobic-polar interface and close to the C-terminus of the next monomer. In the open structure of *So*PIP2;1 tryptophan 35 is repositioned and in addition this conformation is expected to have released the C-terminus, which could have an effect on the environment of tryptophan 35 and therefore quench its fluorescence.

### Mercury Binding Site

**FIGURE 9:**
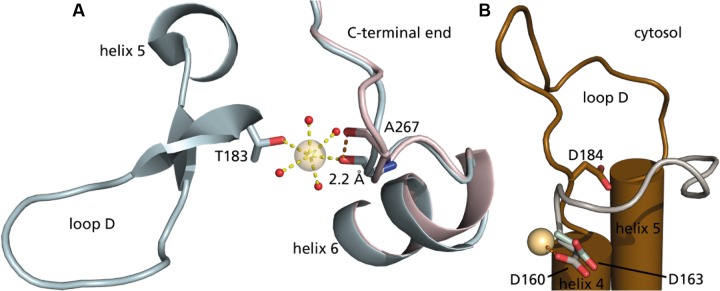
**The second metal binding site in *So*PIP2;1.** **(A)** Cytoplasmic view on two closed structures of *So*PIP2;1, with mercury (PDB ID 4JC6) in blue and without mercury (PDB ID 1Z98) in pink. Alanine 267 is shifted 2.2 Å to coordinate the additional heavy metal site. The heavy atom is further coordinated in this structure by threonine 183 at the beginning of loop D and four water molecules (oxygens in red). **(B)** Comparison of conserved aspartate in the open structure of *So*PIP2;1 (D184, brown, one monomer of PDB ID 2B5F) with aspartates of *At*TIP2;1 (D163, light green, 5I32) and *Bt*AQP1 (D160, gray, 1J4N). The carboxyl group of D160 from the aligned bovine structure is close (2.7 Å) to the heavy metal binding site in 4JC6.

In a mutational study of *So*PIP2;1 by Frick et al. (2013) it was found that mercury activation is independent of the presence of cysteines. They were able to determine a structure at 2.15 Å with bound mercury and cadmium, in a closed conformation. Additional to the expected binding to cysteines and the known Ca^2+^ site, a new metal binding site was reported. It is described as a second binding site for cadmium, but given the comparable size of the cations and similar preference for hexagonal coordination, one could imagine that also mercury has a potential to bind. The metal seen at this site interacts with a carbonyl (A267) from the C-terminal region (**Figure 9A**). The carbonyl of A267 is actually occupying the same space as the carbonyl of A266 in the first closed structure of *So*PIP2;1 (Törnroth-Horsefield et al., 2006), and consequently there is a different position and orientation of preceding residues up to the end of helix 6. This distinctive change in the structure does not change the averaged static view on the C-terminal regions, which overall are located at the interfaces of the monomers in a closed conformation. Still, the binding strength might be reduced and thereby lower the probability to find this conformation in solution. The conformation of the protein when incubated in β-mercaptoethanol after binding mercury does not change within seconds (**Figure 5B**). We expect that mercury binding or chelating is much faster. Thus, the observed time dependence could be a result of the restructuring of the C-terminal end. If the activation is caused by destabilization of the C-terminal stretch, the effect could be reduced in situations where it is already destabilized for other reasons. This is observed, when comparing the permeability of an untagged *So*PIP2;1 WT and the permeability of the same isoform that ends with a c-myc epitope followed by a hexa-his-tag. The basal (i.e., not fully activated) permeability is higher for the tagged protein, seemingly because the closed conformation of the unphosphorylated C-terminal end is less stabilized (data not shown). It should not go unnoted that similar conclusions were drawn before (Nyblom, 2008, Ph.D. thesis, paper III, page 5). When incubated with mercury the specific water permeability, of both the tagged and untagged protein, levels out at approximately the same permeability values. Conclusively, destabilization by binding of mercury overlaps the destabilization caused by the tag, suggesting that the gate is close to its maximum opening probability in the mercury activated state. Frick et al. (2013) suggested an indirect activation of *So*PIP2;1 via a mercury induced change in membrane properties. This possibility cannot be ruled out, but the relatively low EC_50_ of about 4 μM, which is about 10-fold lower than the IC_50_ for *Hs*AQP5 in proteoliposomes with the same lipid composition (Horsefield et al., 2008), and the correlation with the quenching of fluorescence in micelles, would argue for a direct binding to and activation of *So*PIP2;1.

Here, we describe the activation by mercury as a result of moving serine 274 away to leave space for leucine 197 in an open position, to promote the conformation. However, could mercury further stabilize the open conformation? As mercury is positively charged, we looked for a negatively charged residue in the vicinity. There is an aspartate in loop D, which is conserved among water-specific AQPs from both plants and animals. This residue approaches the binding site in the open conformation (**Figure 9B**), but the distance is still too far. Comparison to other water-specific AQPs suggests that the aspartate could come close enough to bind mercury and thereby stabilize the D-loop in an open position. Although the aspartate corresponding to aspartate 184 in *So*PIP2;1 is well conserved not only among PIPs, there is considerable sequence variation at the position aligned to alanine 267 of SoPIP2;1 even within the PIP2 group, making it difficult discern if the proposed activation mechanism is valid for other PIPs. However, the fact that it is the back bone carbonyl of this residue that contributes to the metal binding site, and the general observation that structure is more conserved than sequence, argues that the binding site and the suggested mechanism may be relevant also for other PIP2s of terrestrial plants. It should be noted that the plant plasma membrane has a different lipid composition and is much more complex than the liposomes of *E. coli* lipids used here. Hence, the relevance of the demonstrated mercury activation remains to be shown in a physiological context where indirect effects by mercury via interacting lipids or proteins may override a direct activation.

### Is There a Central Pore?

The functionality of the protein is determined by stabilization of certain conformational states and thereby dependent on the availability of certain interacting residues, not only within a monomer, but also with neighboring monomers. This has already been described for many proteins and underlines that proteins evolve based on their final oligomeric structure. This brings us back to cysteine 69, which is found to be strongly conserved among PIPs and covalently links the monomers but does not add much thermodynamic stability. Interaction studies with PIPs in maize demonstrated that expression rate and localization is also independent of disulfide bond formation (Bienert et al., 2012). Taking only our own results into account, one could suggest the prevention of water leakage through the tetrameric center, but this is inconclusive with the mutational study of maize PIPs measured in oocytes. As the utilized maize PIP2 is very similar to *So*PIP2;1, the most prominent difference between these studies is the protein environment. As several AQP structures, including *So*PIP2;1, have presented a detergent or a lipid molecule in the tetrameric center (Jiang et al., 2006; Horsefield et al., 2008; Frick et al., 2013), one could assume the presence of a lipid molecule at these sites in the native environment to avoid water or even proton leakage. In this study, the protein was heterologously expressed and extracted from the hydrophobic environment by a detergent. These detergent molecules are capable of removing most or all of the lipid molecules bound to the protein. Additionally, the protein–lipid mixture was thoroughly dialyzed to reconstitute this protein in liposomes, potentially removing all detergent molecules. Thus cysteine 69 mutants could leak water through a central pore. As other AQP isoforms without this cysteine have also evolved to be proton tight, there is probably a different reason for the high degree of conservation. We therefore agree with Bienert et al. (2012) that disulfide bonds between monomers result in a higher kinetic stability and thereby stabilize the oligomeric state of the protein, which may comprise certain functions *in planta*.

## Conclusion

In agreement with studies on maize PIPs we show that disulfide bridges stabilize the oligomeric state of SoPIP2;1, but contrary to results on maize PIPs we find that mutations preventing the disulfide bridge increase the permeability. Furthermore, the permeability of both WT and mutant protein can be enhanced by mercury and based on quenching of the intrinsic tryptophan fluorescence by mercury we speculate on a binding site, possibly responsible for both effects of this heavy metal. Mercury has been regarded as general inhibitor for AQPs and therefore used to classify observed *in vivo* phenomena as being “AQP dependent.” The sole possibility that some AQPs under certain circumstances can be activated by mercury puts these conclusions into a new perspective. Mercury is not a physiological relevant ligand to regulate protein function, but presents a tool to study functional properties of the gating mechanism of PIPs and thereby potentially revealing new opportunities to modulate water homeostasis in plants.

## Materials and Methods

### Overexpression and Purification of Cysteine Mutants of *So*PIP2;1

Cysteine mutants of *So*PIP2;1 were generated by using Quickchange site directed mutagenesis kit (Stratagene), using the plasmid pPICZB-*So*PIP2;1 (WT) with C-terminal His-tag as a template (Karlsson et al., 2003). Mutations were confirmed by DNA sequencing (Eurofins MWG operon). Mutants were overexpressed in methylotropic yeast *Pichia pastoris* as previously described (Karlsson et al., 2003) for WT *So*PIP2;1 purification. Similar procedure was followed for membrane preparation and purification of mutated proteins as described previously (Karlsson et al., 2003) with a minor change; the detergent used in the present study is *n*-Octyl-β-D-Glucopyranoside (OG; Affymetrix, O311).

### Electrophoresis

The purified *So*PIP2;1 (WT) was incubated at room temperature for varying time periods, with different concentration of dithiothreitol (DTT) or β-mercaptoethanol to reduce the dimeric form into monomers. After incubation with reducing agent the sample loading buffer (125 mM Tris-HCl, pH 6.8, 20% glycerol, 4% SDS) was mixed with protein sample and further incubated for 10 min at room temperature. To monitored the effect of reducing agent concentration and incubation time, the oligomeric forms of the protein were resolved by SDS-PAGE (12%) and visualized by staining with coomassie brilliant blue R250 (Laemmli, 1970).

In order to compare the SDS-PAGE profile of the WT and mutant proteins (**Figure 3**), the protein was directly mixed with sample loading buffer (as mentioned above) supplemented with 10% β-mercaptoethanol and incubated for 10 min at room temperature, before resolving on SDS-PAGE (12%).

### Circular Dichroism (CD) Spectroscopy

Far-UV CD spectra were measured for the WT *So*PIP2;1 and the mutants using a Jasco J-720 spectrometer (Jasco, Tokyo, Japan). Spectra were recorded at 25–95°C (with 5°C interval) between 250 nm and 190 nm at 20 nm/min as an average of three scans with a response time 8 s and a data pitch of 0.1 nm. Baselines were collected in the same manner on the buffer solution, and spectra were baseline corrected (Galka et al., 2008; Plasencia et al., 2011).

Mean residue ellipticity (MRE, [𝜃]_M_ × 10^-3^ deg cm^2^ dmol^-1^) was calculated by using Eq. (1).

$${\left[\theta \right]}_{M}=M\times \theta /\left(10\times 1\times c\times n\right)$$

where M is the molecular weight of protein (e.g., 32512 g/mol), 𝜃 is the measured ellipticity in millidegrees, l is the cell path length, c is the concentration in [g/l], and n is the number of residues (303).

The MRE at 222 nm was plotted over temperature. For curve fitting, following Boltzmann sigmoidal equation was used:

$$Y_{obs}=Y_{native}-\frac{Y_{denatured}-Y_{native}}{1+e^{T_{1/2}-\frac{T}{m}}}$$

Where Y_obs_ is the MRE, T_1/2_ is the temperature at which MRE is halfway between native and denatured state, m is the slope of the curve. Data was analyzed using Prism (Graphpad software, Inc.).

### Reconstitution into Liposomes

*E. coli* POLAR lipids (Avanti) provided in chloroform were dried with N_2_ for 4 h and kept at -20°C until use. For reconstitution the lipids were solubilized with 10% OG in dialyze buffer (20 mM Tris pH 8, 100 mM NaCl, 0.003% NaN_2_, 2 mM DTT) for concentration of 4 mg/mL and aliquoted. Proteins were added and solution was mixed thoroughly. Lipid–protein mix was diluted with dialyze buffer to 2 mg/mL lipids and 66 μg/mL proteins (LPR30) and dialyzed using a membrane with 6–8 kDa cut-off (Spectrum Laboratories) against dialyze buffer for 7 days at room temperature.

### Water Conductivity

Liposomes were extruded 11 times with a pore size of 200 nm (Avestin) and their resulting average radius was determined by dynamic light scattering DLS (Malvern Zetasizer). Samples were diluted with dialyze buffer (with or without mercury) to 0.2 mg/mL lipids. To show that activation of the protein is reversible, a sample incubated for 30 min with 1 mM HgCl_2_, 2 mM β-mercaptoethanol was added and incubated at least 30 min further prior to the activity assay. Water transport activity was measured by stopped-flow with a hyper-osmolar gradient of 100 mM sorbitol using Hi-Tech stopped-flow device at a volume of 150 μL per shot. Rise in scattering upon shape change due to water transport out of the vesicles was observed at 90° angle at a wavelength of 500 nm. Unless mentioned otherwise, single exponential functions were fitted to 10 to 17 individual curves by the software Kinetic Studio (TgK Scientific Limited 2010). Total water permeability *P*_f_ is used to calculate individual *p*_f_-values by multiplying relative *P*_f_ [*P*_f_ (proteoliposome) – *P*_f_ (control liposomes)] with the surface area of the liposome and dividing with the number of monomers per vesicle.

$$P_{f}=k\cdot \frac{V_{0}}{A\cdot V_{w}\cdot c_{out}}$$

$$P_{f}=\frac{(P_{f}-P_{f,control})\cdot A}{\#monomers}$$

Protein concentration was analyzed by Western-blot using tetra-His antibody, and vesicle concentration was calculated assuming no lipid loss during dialysis and an area of 0.52 nm^2^ per lipid molecule (for a monolayer). To construct a dose response curve of mercury activation, single exponential rates from 10 to 16 stopped-flow measurements were averaged per samples exposed to different mercury concentration; each average is presented with their standard deviations. Curve fitting was done employing a Hill-function.

### Statistical Analyses

The significance was analyzed in Prism (Graphpad software, Inc.), using the unpaired *t*-test with Welch’s correction and two-tailed *P*-values.

### Tryptophan Fluorescence

Fluorescence was measured with His-tag purified and desalted protein in Buffer A supported by 0.8% OG. Monochromatic light at a wavelength of 280 nm was used for excitation while scans were done from 300 to 380 nm at a speed of 100 nm/min and a data pitch of 0.5 nm. Fifteen scans were accumulated to reduce noise. For kinetic experiments, mercury chloride to a final concentration of 200 μM was added and briefly mixed before starting scans. First, single records were done with a delay of 1 s, resulting in one plot every 49 s. The averages of five data points were used to determine height of the curve maximum. After incubating for 25 min, another accumulated spectrum was recorded. Sequentially, β-mercaptoethanol to a final concentration of 1 mM was added to reverse the binding, followed by the same measurement procedure as before.

For dose-response experiments, the sample was pre-incubated with mercury chloride at room temperature for 15 min before recording the fluorescence between 310 and 400 nm. Three scans were accumulated to reduce the noise and the measurement was repeated tree times. The emission data at 335 nm were fitted with four parameters to sigmoidal dose response equation using Prism version 6.00 (GraphPad Software, La Jolla, CA, USA) to estimate the EC_50_ value.

## Author Contributions

AK and SS contributed equally to this work, did all the technical work, and analyzed the data. PK and UJ conceived the project. AK drafted the manuscript and all authors contributed to writing of the final article.
