# Corrigendum: Increased Permeability of the Aquaporin *So*PIP2;1 by Mercury and Mutations in Loop A

**DOI:** 10.3389/fpls.2016.01888

**Published:** 2016-12-19

**Authors:** Andreas Kirscht, Sabeen Survery, Per Kjellbom, Urban Johanson

**Affiliations:** Department of Biochemistry and Structural Biology, Center for Molecular Protein Science, Lund UniversityLund, Sweden

**Keywords:** aquaporin, water channel, major intrinsic protein, *Spinacia oleracea*, tryptophan fluorescence

In our Original Research article it was incorrectly stated that cadmium and mercury have a similar preference for hexagonal coordination. Although both cadmium and mercury can bind at octahedral (not hexagonal) coordination sites their coordination preferences are not similar (Rulisek and Vondrasek, 1998). Hence, the fourth sentence under the subheading “Mercury Binding Site” in the Discussion should read: “It is described as a second binding site for cadmium, but given the comparable size of the cations and that both are capable of forming an octahedral coordination geometry (Rulisek and Vondrasek, 1998), one could imagine that also mercury has a potential to bind.” The authors apologize for the mistake. This error does not change the scientific conclusions of the article in any way.

## Conflict of interest statement

The authors declare that the research was conducted in the absence of any commercial or financial relationships that could be construed as a potential conflict of interest.
