# Supplementary material for: Increased Permeability of the Aquaporin SoPIP2;1 by Mercury and Mutations in Loop A
Source: Front Plant Sci. 2016 Aug 30;7:1249. doi: 10.3389/fpls.2016.01249 (PMC5004352; doi:10.3389/fpls.2016.01249)
Supplement: Supplementary file 1 [file Data_Sheet_1.PDF]

Supplementary Material

Increased Permeability of the Aquaporin *So*PIP2;1 by Mercury and Mutations in Loop A

Andreas Kirscht†, Sabeen Survery†, Per Kjellbom and Urban Johanson *

† Shared first authors, names in alphabetical order

*** Correspondence:** Urban.Johanson@biochemistry.lu.se

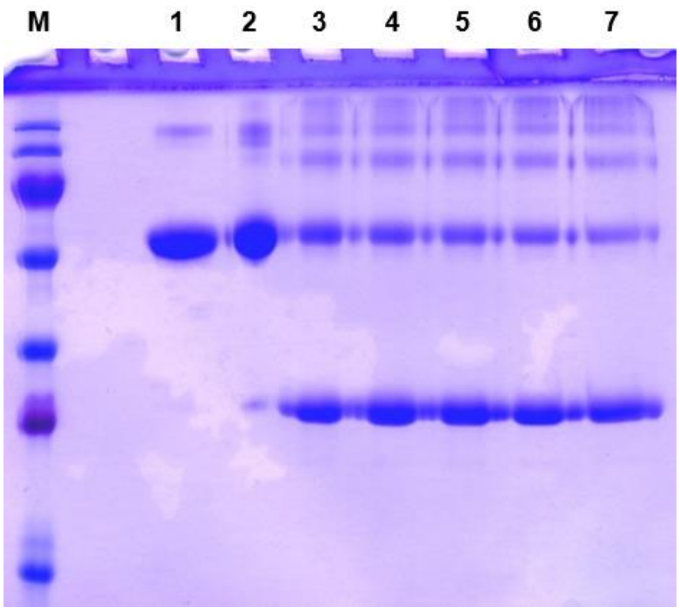

Supplementary Figure 1. Reduction of *So*PIP2;1 by DTT in the presence of 8 M urea. Coomassie-stained SDS-PAGE gel with 8 M urea showing samples originally maintained in PBS and 1% OG, diluted 20-fold in: buffer (PBS, 1% OG), water or varying concentrations of DTT supplemented with 8 M urea. All samples were incubated at room temperature for one hour. With increasing concentration of DTT the dimeric band becomes weaker relative the monomeric band, but is not completely abolished even after incubation in 300 mM DTT and 8 M urea. 1 = *So*PIP2;1 dissolved in PBS (1% OG), 2 = *So*PIP2;1 dissolved in water, 3 = 100 mM DTT and 8 M urea, 4 = 150 mM DTT and 8 M urea, 5 = 200 mM DTT and 8 M urea, 6 = 250 mM DTT and 8 M urea, 7 = 300 mM DTT and 8 M urea. Equal amounts of protein were loaded in all lanes. M = molecular weight marker (from top 250, 130, 100 and 70 not separated, 55, 35, 25, 15 and 10 kDa).

Supplementary Table 1. Single exponential rates for traces presented in Figure 4A used to calculate p_f values presented in Figure 4B.

Sample	Average rate (s^{-1})	Standard error of the mean (s^{-1})	p_f (10^{-14} cm^3 s^{-1})	Standard deviation (10^{-14} cm^3 s^{-1})
Empty liposomes	6.47	0.02		
*So*PIP2;1	14.61	0.12	1.61	0.09
*So*PIP2;1 + $HgCl_2$	39.26	0.92	3.41	0.24
C69S	22.07	0.17	2.22	0.10
C69S + $HgCl_2$	27.93	0.37	6.02	0.69
C69A	28.30	0.26	3.64	0.23
C69A + $HgCl_2$	35.32	0.48	8.02	0.60

Supplementary Table 2. *P* values for pairwise comparisons of data presented in Fig. 4B, unpaired t-test with Welch's correction.

Sample	*So*PIP2;1 + $HgCl_2$	C69S	C69S + $HgCl_2$	C69A	C69A + $HgCl_2$
*So*PIP2;1	<0.0001	0.0003	0.0001	<0.0001	<0.0001
*So*PIP2;1 + $HgCl_2$		0.0007	0.0043	**0.4984**	<0.0001
C69S			0.0003	0.0001	<0.0001
C69S + $HgCl_2$				0.0075	0.0424
C69A					<0.0001

Supplementary Table 3. *P* values for pairwise comparisons of data presented in Fig. 6A, using unpaired t-test with Welch's correction.

Sample	Empty + $HgCl_2$	Empty + $HgCl_2$ + β-mercapto-ethanol	*So*PIP2;1	*So*PIP2;1 + $HgCl_2$	*So*PIP2;1 + $HgCl_2$ + β-mercapto-ethanol
Empty liposomes	<0.0001	<0.0001	<0.0001	<0.0001	<0.0001
Empty + $HgCl_2$		**0.1361**	<0.0001	<0.0001	<0.0001
Empty + $HgCl_2$ + β-mercaptoethanol			<0.0001	<0.0001	<0.0001
*So*PIP2;1				<0.0001	**>0.9999**
*So*PIP2;1 + $HgCl_2$					<0.0001

Supplementary Table 4. Numerical values for values presented in Fig. 6A

Sample	Average rate (s^{-1})	Standard deviation (s^{-1})
Empty liposomes	5.37	0.05
Empty + $HgCl_2$	7.39	0.04
Empty + $HgCl_2$ + β-mercaptoethanol	7.29	0.05
*So*PIP2;1	14.69	0.61
*So*PIP2;1 + $HgCl_2$	39.99	2.69
*So*PIP2;1 + $HgCl_2$ + β-mercaptoethanol	14.61	0.45
